# Anatomy of the levator claviculae, with an overview and a literature survey

**DOI:** 10.1007/s12565-012-0148-8

**Published:** 2012-08-26

**Authors:** Toru Odate, Masataka Kawai, Kazuki Iio, Satoshi Funayama, Haruo Futamata, Sen Takeda

**Affiliations:** Department of Anatomy and Cell Biology, University of Yamanashi Interdisciplinary Graduate School of Medicine and Engineering, 1110 Shimo-Kateau, Chuo, Yamanashi 409-3898 Japan

**Keywords:** Anomaly, Levator claviculae, Ontogeny, Phylogeny, Primates

## Abstract

We report here an anatomical study of the levator claviculae discovered during an anatomical dissection course for medical students. The muscle was identified on the left side, and followed a typical topography to previous detections, originating from the transverse process of the fourth cervical vertebra and attaching to the upper facet of the middle part of the clavicle. Innervation to this muscle came from both the third and fourth rami of the cervical spinal nerves. Blood supply to the muscle could not be identified clearly. In this report, we undertook a comprehensive literature survey of this muscle dating back ca. 170 years, and attempted to ascertain the phylogenic and ontogenetic explanations for the development of this muscle.

## Introduction

The levator claviculae is a muscular variation in humans located in the posterior cervical triangle of the neck (Gruber 1876; Nagashima et al. 1989; Tomo et al. 1994; Leon et al. 1995). It usually originates from the transverse process of the upper cervical vertebrae, and attaches to the lateral aspect of the clavicle (Eisler 1912; Koshy et al. 2005). While some articles in the literature have reported a tendency of this muscle to appear on the left side (Rubinstein et al. 1999; Capo and Spinner 2007), this is not a strict rule (Holibková and Machálek 1998; Feigl and Pixner 2011). In primates other than humans (Gibbs et al. 2002), this muscle is always present bilaterally. Although the nerve supply usually comes from the cervical plexus, we do not know exactly which nerve branch innervates this muscle (Rodríguez-Vázquez et al. 2009). There are currently some hypotheses concerning its embryological origin: sternocleidomastoid (Rodríguez-Vázquez et al. 2009), trapezius (Parsons 1898), ventrolateral muscle primordium of the neck (Leon et al. 1995), anterior scalene (Gruber 1876), and longus colli (LCO) (Tomo et al. 1994).

The incidence of this muscle is reported to be 2–3 % (Wood 1870; Testut 1884; Le Double 1897; Rubinstein et al. 1999). However, Loukas and colleagues, after dissecting approximately 2,000 cadavers, reported that the previously described figures are somewhat overestimated, as they found only two cases in their cohort (Loukas et al. 2008). Moreover, despite a seeming paucity of documented cases, when we pushed our search back to the 1800s, we identified many more published case reports (Macalister 1866; Davies-Colley et al. 1873; Knott 1880). Although its presence is unusual in humans, the levator claviculae exists unequivocally in almost all mammals (Wood 1870). However, its origin and insertion vary greatly among species. Owing to these variations, this muscle is known by many different terms among animals, which adds to the confusion (Eisler 1912). As such, this paper aims to provide a comprehensive summary of the levator claviculae, and offers a comparative consideration of its ontogeny and phylogeny, drawing from previous major reports on this muscle.

## Materials and methods

We discovered the presence of the levator claviculae on the left side of the cadaver of an 89-year-old female who died from heart failure. It was discovered during a dissection for the medical students of the laboratory course at the University of Yamanashi in 2011.

The cadaver was perfused with 10 L of 3 % formalin for 48 h by an electric pump via the right femoral artery. After perfusion, the cadaver remained untouched for one week to allow penetration of the fixative. Subsequently, the fixed cadaver was placed into a chamber to replace the fixative with 70 % ethanol. Approximately one month after immersion, the cadaver was used for dissection. All protocols for receiving donated human cadavers, as well as the procedures for dissection in the laboratory course for medical students, were approved by the donor and the ethical committee at the University of Yamanashi.

Using the available relevant literature, dating back to the 1800s, we re-examined the ontogenetic and phylogenic aspects of the levator claviculae.

## Results

**Fig. 1:**
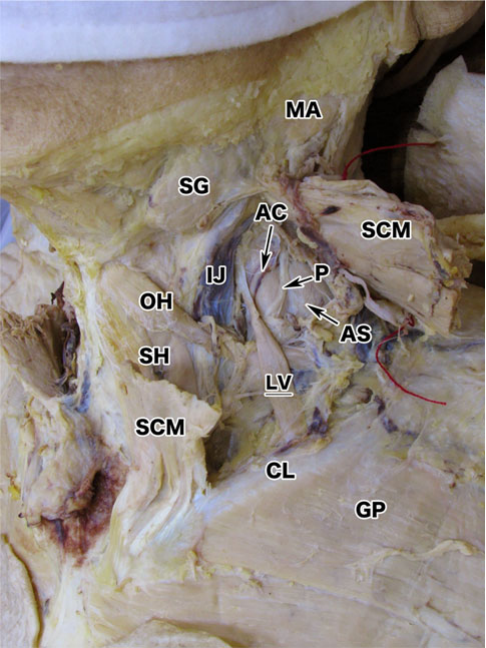
Overview of the levator claviculae. The levator claviculae (*LV*) was found unilaterally in the left lateral cervical region. Its insertion was in the middle of the left clavicle (*CL*). This panel does not clearly show the origin of the muscle. It was located posterior to the sternocleidomastoid (*SCM*); anterior to the omohyoid (*OH*), the ascending cervical artery (*AC*) and the phrenic nerve (*P*); and lateral to the internal jugular vein (*IJ*). The phrenic nerve and the AC run anterior to the anterior scalene (*AS*), as usual. The SCM has been cut in the middle and reflected in a cranial and caudal direction. The mandibular angle (*MA*), the submandibular gland (*SG*), the sternohyoid (*SH*), and the greater pectoral (*GP*) are also shown

In our case, the levator claviculae was identified as a flat and thin muscle (LV, Fig. 1). It spanned 55 mm in length from its origin to insertion, and was 9 mm at its widest part. It was located posterior to (and almost completely overridden by) the sternocleidomastoid. We found the brachial plexus, the anterior scalene (AS) and the omohyoid (OH) situated dorsal to this muscle, and the internal jugular vein (IJ) and the sternohyoid (SH) medial to this muscle (Fig. 1). The ascending cervical artery (AC, Figs. 1, 3) and the phrenic nerve (P, Figs. 1, 3) ran posterior to the levator claviculae. Although the phrenic nerve appeared to be located behind the anterior scalene (Fig. 3), the original position of the phrenic nerve was on the ventral surface of the anterior scalene, running obliquely from superior-lateral to inferior-medial (Fig. 1). However, as the dissection progressed, the phrenic nerve was artificially placed dorsal to the anterior scalene to facilitate the dissection. The ascending cervical artery was not derived from the thyrocervical trunk but from the transverse cervical artery.

**Fig. 2:**
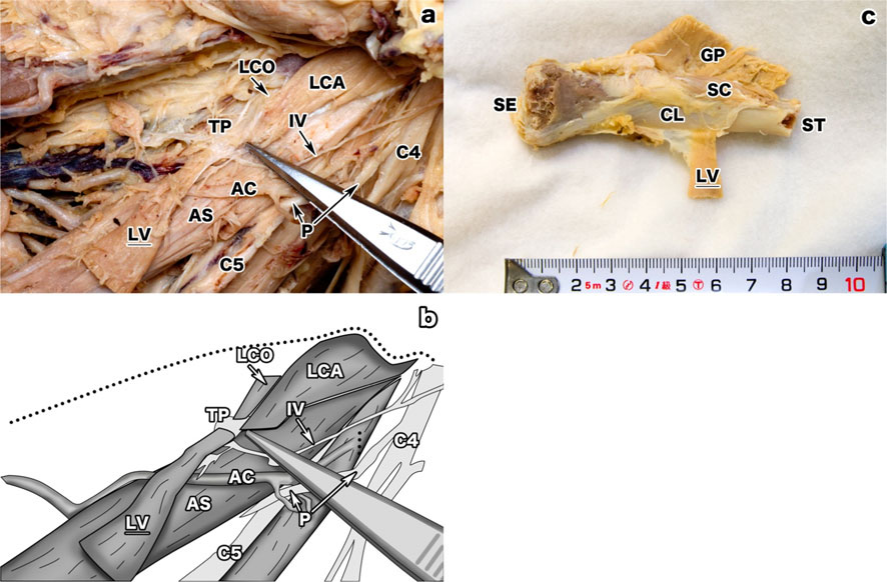
Enlarged view of the origin and insertion of the levator claviculae. **a** The enlarged superior part of the levator claviculae (*LV*). The origin of the levator claviculae was identified as the anterior tubercle of the C4 transverse process (*TP*), as indicated by a pair of tweezers. The longus colli (*LCO*) and the longus capitis (*LCA*) also inserted onto the process. The LV was located anterior to the ascending cervical artery (*AC*), the phrenic nerve (*P*), and the anterior scalene (*AS*). Although the phrenic nerve seemed to be situated behind the anterior scalene, this state was intentionally made during the dissection to show the LV clearly. Originally, before starting the dissection deep into the neck, the phrenic nerve was located anterior to the anterior scalene normally (Fig. 1). The C5 nerve (*C5*) was located between the anterior and middle scalene. The C4 nerve (*C4*) and the innervation (*IV*) of the levator claviculae are also shown. **b** An illustration of **a**. This panel emphasizes the origin of the LV, the periphery of the C4 transverse processes, and the innervating branch (*IV*). The *dotted line* indicates the border of cervical midline mass such as thyroid gland and trachea. **c** A magnified image of the levator claviculae (*LV*) attached to the posterior surface of the clavicle (*CL*). The insertion of the levator claviculae was onto the middle of the clavicle, about 50 mm lateral to the sternal end (*SE*). No other muscles shared this insertion point. The greater pectoral (*GP*), the subclavius (*SC*), and the stump of the clavicle (*ST*) are shown

The levator claviculae originated from the anterior tubercle of the C4 transverse process as a tendinous slip, which made contact with the LCO and the longus capitis (LCA) (Fig. 2). However, while the levator claviculae ran inferiorly, the LCO and the longus capitis ran in superior-medial and superior-lateral directions, respectively. The insertion of the levator claviculae was made on the posterior upper surface of the middle of the clavicle (CL) as a muscular fascicle, and was approximately 18 mm from the insertion point of the sternocleidomastoid on the clavicle (Fig. 2). Unlike its origin, where the convergence of three muscles was observed, there were no other muscles sharing this insertion point.

**Fig. 3:**
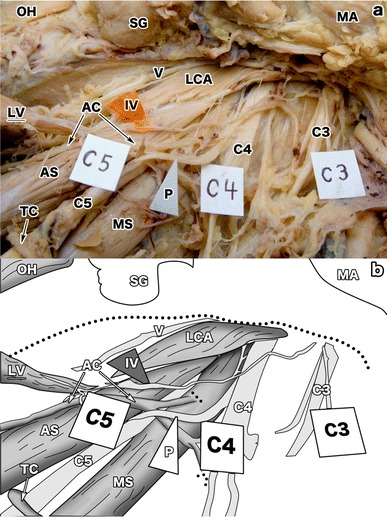
The innervation of the levator claviculae. **a** An overview of the innervation (*IV*) of the levator claviculae (*LV*). The innervation was from the C3 (*C3*) and the C4 (*C4*) nerves, running caudally along the anterior scalene (*AS*), the ascending cervical artery (*AC*), and the C5 (*C5*) nerve. The nerve was situated lateral to the longus capitis (*LCA*), medial to the phrenic nerve (*P*). Although the phrenic nerve and the transverse cervical artery (*TC*) seemed to be located behind the anterior scalene, this was an artifact generated after the dissection. Originally, they were located anterior to the anterior scalene as usual (see Fig. 1). The C5 nerve, the omohyoid (*OH*), the submandibular gland (*SG*), the mandibular angle (*MA*), and the vagal nerve (*V*) are also indicated. **b** An illustration of **a**. This panel emphasizes the innervation with special references to its route and the innervated tissue

The innervation of this muscle was derived from the C3 and C4 spinal nerves (Fig. 3). The C3 and C4 spinal nerves anastomosed to form a single branch which crossed over the C5 nerve and entered the muscle dorsally near its origin (Fig. 3). We could not identify any arterial supply to this muscle clearly. Collectively, the levator claviculae reported herein appears to share common features of previous reports.

## Discussion

### Common features of the levator claviculae: a literature review

The levator claviculae is regarded as a supernumerary muscle in humans, but a normal constituent of the neck and shoulder girdle in most mammals. Therefore, this muscle may offer an interesting evolutionary perspective regarding the origin of human beings. As far as we know, in the past 170 years, there have been approximately 30 reports detailing the existence of the levator claviculae in humans (Table 1). By arranging a compendium of these papers, we extracted several central features of this muscle. Concerning laterality, which may have a significant meaning in terms of ontogeny, there appears to be no tendency for the levator claviculae to appear on a specific side, even though some reports claimed that it is more often discovered on the left side (Rubinstein et al. 1999; Capo and Spinner 2007; Loukas et al. 2008). Moreover, as shown in Table 1, the occurrence of this muscle on the right side is not rare (Gruber 1876; Leon et al. 1995).

**Table 1:** Summary of bibliographies on the levator claviculae

Author	Age (years)	Sex	Laterality	Origin	Insertion	Innervation	Note
Theile (1843)	ND	Male	Right	C4, C5	Middle of the CL	ND	The LV crossed over the omohyoid
M'Whinnie (1846)	ND	ND	ND	Upper cervical vertebrae	CL	ND	M'Whinnie repoted two cases in this report
Wood (1864)	ND	Male	Bilateral	C3, C4	Lateral third of the CL	ND	
Macalister (1866)	ND	Female	ND	ND	Lateral third of the CL	ND	
Flower and Murie (1867)	Adult	Female	ND	Levator scapulae	Serratus anterior	ND	Authors considered this variation as the LV
Wood (1870)	ND	See note	See note	ND	ND	ND	Wood reported six cases in this article (five males and one female). In three the LV was on both sides, in two it was on the left side
Macalister (1871)	ND	ND	ND	Lower cervical vertebrae	See note	ND	Macalister found three patterns of insertion. They are acromion, lateral third of the CL, and the TZ
Davies-Colley et al. (1873)	ND	ND	ND	Upper cervical vertebrae	Lateral CL	ND	
Gruber (1876)	Child	Male	Right	C6	Medial of the CL	ND	
Brown (1880)	ND	ND	Unilateral	C1, C2	Middle of the CL	C2	There was no mention of which side the LV appeared on
Knott (1880) 1	ND	ND	ND	C3, C4	Middle of the CL	ND	
Knott (1880) 2	ND	ND	ND	C6	CL	ND	The insertion of the muscle was blended with one of the TZ
Walsham (1881)	ND	ND	ND	C1	Medial of the CL	ND	
Testut (1884)	ND	Female	Left	C3	Acromial end	ND	The “cléido-omo-transversaire” is a more appropriate nomenclature according to the author
Le Double (1897)	ND	ND	ND	C1,C2	Acromial end	C4	
Eisler (1912)	ND	ND	Left	C1,C2	Acromial end	ND	
Patten (1934)	71	Male	Left	C1, C2, C3	Acromial end	C3, C4	
Nagashima et al. (1989)	69	Male	Bilateral	C6	CL	C2, C3 and ansa cervicalis	The authors also found the cleido-occipitalis in the same cadevar. It was situated only in the left side
Tomo et al. (1994)	87	Male	Left	C6	Middle third of the CL	C5	The superior root of the ansa cervicalis was on the ventral side of the LC and the inferior root was on the dorsal side
Leon et al. (1995)	65	Male	Right	C2	Middle third of the CL	C4	The omohyoid had two heads of origin
O’Sullivan and Kay (1998)	36	Male	Left	TZ	Medial aspect of the CL	ND	The muscle originated from the TZ directly
Holibková and Machálek (1998)	Adult	Male	Bilateral	C1, C2	Lateral CL	C2, C3, C4	
Koshy et al. (2005)	67	Male	Bilateral	C1, C2	Lateral third of the CL	C2, C3, C4	The muscle inserted into the CL together with fibers of the TZ
Capo and Spinner (2007)	72	Male	Left	C2	Lateral CL	C2	
Loukas et al. (2008)	78	Male	Left	C3, C4	Lateral third of the CL	C5, C6	
Natsis et al. (2009)	65	Male	Right	C3, C4, C5	Acromial end	C4	
Rodríguez-Vázquez et al. (2009)	71	Male	Right	C1	Middle third of the CL	Supraclavicular nerve	There was a loop of nerves surrounding the LV. It was made by transverse cervical nerve and the greater auricular nerve
Feigl and Pixner (2011)	88	Female	Right	C1	SCM	C3	The muscle separated from the SCM directly
Present case	89	Female	Left	C4	Middle of the CL	C3, C4	

This table outlines the cardinal features (author, age, sex, laterality, origin, insertion, and innervation) of the previous reports on the levator claviculae. Other important information has been included as a note

*ND* not determined, *CL* clavicle, *LV* levator claviculae, *SCM* sternocleidomastoid, *TZ* trapezius

There are varied reports as to the origin of the muscle, ranging from C1 to C6. One intriguing exception was reported by Flower and Murie (1867), wherein an additional slip of muscle separated from the levator scapulae and reached to the serratus anterior. Moreover, O’Sullivan and Kay (1998) reported another unusual variant of the levator claviculae that originated directly from the trapezius; there is debate as to whether these variants should be termed levator claviculae.

In most cases, the levator claviculae insertion was identified on the clavicle, although a range of positions have been reported. We divided these insertion points into three groups: medial, middle, and lateral parts of the clavicle. As a rule, the levator claviculae inserts onto the middle or lateral clavicle. However, insertion onto the sternocleidomastoid (Feigl and Pixner 2011) and the serratus anterior (Flower and Murie 1867) has been noted.

Regarding its innervation, we could only identify explicit descriptions in reports appearing after the 1900s. While the pattern of innervation varies between reports, the branches of the ventral nerve from the cervical plexus are linked predominantly to the innervation of the levator claviculae.

### What is the ontogenetic origin of the levator claviculae?

**Fig. 4:**
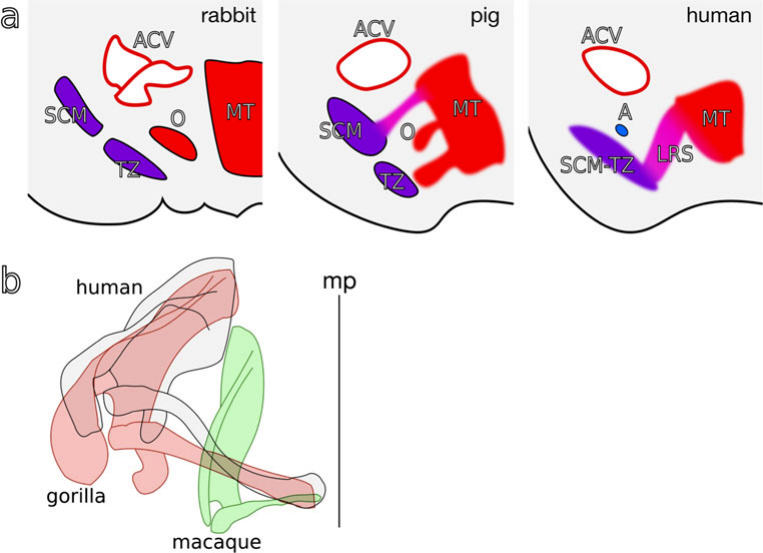
Hypothesis for the embryonic and phylogenic development of LV. **a** Illustrations showing transverse sections of rabbit, pig, and human embryos at the neck according to McKenzie (1955). For the rabbit and human embryos, we referred to the histological sections reported therein. For the pig embryo, locations for each muscular component were drawn according to the description by McKenzie (1955). *Left*: the rabbit embryo at day 14; crown to rump length (*CRL*) 10.5 mm. The sternocleidomastoid-trapezius (*SCM*-*TZ*), pre-muscle myotome (*MT*), and anterior cardinal vein (*ACV*) are indicated. A discrete anlage of the omocervicalis (*O*), a homolog of the levator claviculae, lies between the myotome and the SCM-TZ pre-muscle mass. *Middle*: an illustration showing an imaginary transverse section of a pig embryo with a CRL of 12 mm. The differentiating trapezius (*TZ*) and the sternocleidomastoid (*SCM*) are indicated. In pig, parts of the myotome extend to the SCM and TZ, both of which are composed of branchial and myotomal muscle fibers. The omocervicalis is derived from the same myotome. Therefore, the anlage of omocervicalis is not well demarcated from the surrounding structures. *Right*: the human embryo with a CRL of 9 mm. Humans usually lack the omocervicalis. Instead, a muscle sheet giving rise to the levator scapulae, rhomboideus, and serratus anterior (*LRS*) develops from the myotome, and some of the muscle fibers contribute to the myogenesis of the SCM-TZ. The accessory nerve (*A*) innervating the SCM-TZ locates superior-medial to the SCM-TZ. **b** A Birds-eye camera lucida view of the shoulder girdle of the macaque (*green*), gorilla (*red*), and human (*gray*). The distance between the acromion and the trunk elongated with changes in lifestyle from arboreal to terrestrial (i.e., from the macaque to gorilla to human). In addition, the angle between the clavicle and the median plane (mp, Fig. 4b) changed. These changes resulted in a broader shoulder and a more outward-oriented glenoid fossa (colour figure online)

The embryologic origin of the levator claviculae has been a source of disagreement. Many hypotheses have been made based on phylogenic and topological considerations. As there is no experimental report examining the anlage of the levator claviculae by the deletion of anlage or specific genes, we cannot draw a definitive conclusion on the basis of developmental biology. To our knowledge, McKenzie (1955) made the most of embryo specimens by cutting serial sections and tried to understand the ontogeny of the levator claviculae in rabbit, pig, and human. Furthermore, he deepened his study of human embryos by carefully observing serial sections at various stages of development (Carnegie stages 14–18), and proposed some ideas on the ontogeny of the levator claviculae, with special references to the ontogeny of the sternomastoid (McKenzie 1962). According to his description, the anlage of the omocervicalis (levator claviculae) in rabbit appears as a discrete mass between the cranial half of the sternomastoid-trapezius (SCM-TZ, Fig. 4a) mass and myotome (MT, Fig. 4a). This discrete anlage of the omocervicalis developed independently, and settled onto the scapula instead of the clavicle (this muscle is therefore denominated the “omo”-cervicalis, in lieu of the levator claviculae). In pig, this discrete mass becomes somewhat undefined mesenchymal condensation linking the SCM-TZ and MT (McKenzie 1955). In human, this mesenchymal mass joins to the SCM-TZ without demarcation, especially alongside the cervical nerves (McKenzie 1955, 1962), resulting in the SCM-TZ muscle having both branchiogenic and somitic origin. Based on these comparative observations, McKenzie concluded that the anlage of the omocervicalis bona fide lies between the cranial half of the SCM-TZ mass and myotome (McKenzie 1955). McKenzie regarded this myotome as a sheet of muscle giving rise to the levator scapulae, rhomboid, and serratus anterior by citing Giebel (1874). However, technically, Giebel (1874) only insisted on the possibility that the levator claviculae arose from the common anlage of the levator scapulae in his report. Integrating the hypotheses proposed by McKenzie and Giebel, it is plausible that the myotome is destined to be part of the SCM-TZ and the levator scapulae divides into a discrete muscle bundle (i.e., the levator claviculae). This idea seems to reconcile with other cases where the levator claviculae directly originated from the trapezius (O’Sullivan and Kay 1998) and from the sternocleidomastoid (Feigl and Pixner 2011). Moreover, this connection between the cervical myotome and the brachiogenic trapezius and sternocleidomastoid might explain why these two neck muscles derived from the brachial arch have two sources of innervation: spinal nerves and the accessory nerve (McKenzie 1955).

In addition to the shared embryological origin of the levator scapulae and the levator claviculae, we can point out anatomical characteristics shared by these two muscles. First, the innervation of the levator claviculae is similar to that of the levator scapulae. Both muscles are mainly innervated by C3 and C4 and sometimes receive the branch of C5. Besides their innervations, their topographies are similar; these two muscles originate from the upper cervical vertebrae. These muscles generally both insert onto the scapula in mammals except for humans.

There are six other candidate muscles proposed hitherto: sternocleidomastoid (Rodríguez-Vázquez et al. 2009); trapezius (Parsons 1898); sternocleidomastoid and trapezius (Fasel et al. 1994; Holibková and Machálek 1998); LCO (Tomo et al. 1994); ventrolateral muscle primordium of the neck, viz. the scalene, anterior vertebral muscles and infrahyoid muscles (Leon et al. 1995; Koshy et al. 2005); and anterior scalene (Theile 1843; Gruber 1876). Rodríguez-Vázquez et al. (2009) defined the origin of the levator claviculae as the sternocleidomastoid from their observation. According to their assumption, the great auricular nerve and the transverse cervical nerve, which existed between the two muscles in their case, caused the separation of the levator claviculae from the anlage of the sternocleidomastoid. On the other hand, Parsons (1898) regarded this muscle as part of the trapezius muscle sheet, which has a deeper attachment site at the transverse process than the trapezius “proper.”

More recently, Holibková and Machálek (1998) hypothesized that the levator claviculae is a hybrid of the trapezius and the sternocleidomastoid. They suggest that these two muscles derive from both the brachial and somitic anlage, which may serve as the source of supernumerary muscle bundles, one of which is the levator claviculae. Another very curious idea, proposed by Tomo et al. (1994), holds that the upper insertion of the LCO rotated on its attachment to the transverse process and settled onto the clavicle. Thus, according to this theory, the levator claviculae is a derivative of the LCO. Although it is definitely a unique idea, this assumption appears to be based on the topography of the muscle in adults, rather than embryological development. Overall, these hypotheses conceptually overlap one another, and there is no experimental evidence supporting them, so no definitive conclusion can be drawn at this moment.

### Comparative anatomy of the levator claviculae among mammals

From a comparative anatomical point of view, the levator claviculae is ubiquitous in mammals (Wood 1870; Parsons 1898), although several differences exist in its morphology and nomenclature among species. For example, a variety of names have been assigned to this muscle, chiefly according to their origin and insertion: the cleidoatlanticus (Rodríguez-Vázquez et al. 2009), the acromio-trachélien (Gruber 1876), the omo-cleido-transversarius (Giebel 1874), the omocervicalis (McKenzie 1955), the atlantoscapularis anterior (Kajiyama 1970), the cleido-cervicalis (Gruber 1876), the cléido-omo-transversaire (Testut 1884), and the omo-trachelian (Parsons 1898). Quite confusingly, these synonyms are often used interchangeably in various contexts.

A common muscle in primates is a possible homolog of the levator claviculae: the atlantoscapularis anterior. In *Macaca cyclopis* (Formosan rock macaque), the atlantoscapularis anterior originates from the transverse process of the atlas and inserts broadly from the acromion to the lateral one-half to one-third of the upper margin of the spine of the scapula. This muscle is innervated by the third and fourth cervical nerves (Kajiyama 1970). While the origin on the transverse process of the atlas has been conserved in some primates (Stewart 1936), the insertion varies between species (Kajiyama 1970). In gorillas and some chimpanzees, the levator claviculae originates from the transverse process of the atlas and inserts onto the lateral half of the clavicle (Stewart 1936). Note that clavicular attachment is also seen in bats (Parsons 1898).

In macaques, the clavicle is stunted (Fig. 4b). On the other hand, gorillas and humans have a well-developed clavicle (Fig. 4b). As such, the muscle cannot reach the scapula, but instead inserts onto the clavicle. This explanation is based on the distance from the median plane of the body to the acromion (Fig. 4b). Considering the differences in this distance and the angle between the clavicle and the median plane among various species, it is plausible to speculate that the insertion moved from the acromion to the clavicle during evolution (compare macaque with human in Fig. 4b, Parsons 1898; Kajiyama 1970). In addition, clavicular translocation of the levator claviculae may be related to the upright posture and free locomotion of the upper arm in humans.

The levator claviculae is generally thought to be involved in the elevation of the clavicle and lateral flexion of the upper neck region of the spine (Holibková and Machálek 1998), and may act as an auxiliary muscle for respiration (Tomo et al. 1994). However, a previous report (Aydoğ et al. 2007) demonstrated a case of a gymnast with a well-developed levator claviculae. This implies that this muscle functions as a synergist of the trapezius and the serratus anterior when the arm is raised above the horizontal plane of the shoulder. We surmised that the levator claviculae has disappeared during evolution in parallel with the significant decrease in the need to raise the arm higher than the shoulder, as well as with the development of the serratus anterior and the morphological rearrangement of the neck and shoulder girdle (Fig. 4b).

## Clinical implications

In the clinic, the levator claviculae has the potential to be misidentified as a cyst, an arterial aneurysm, a neurofibroma, metastasis, a lymphadenopathy (Rüdisüli 1995; Rosenheimer et al. 2000), the sternocleidomastoid (Feigl and Pixner 2011), or a thrombosed vein (Rubinstein et al. 1999) in imaging diagnostics. From another standpoint, the levator claviculae has the potential to cause thoracic outlet syndrome, a rare condition that involves compression at the superior thoracic outlet, leading to pain, arm discoloration, and tingling, among other symptoms (Aydoğ et al. 2007). As such, it is important for surgeons and radiologists to be aware of this variation (O’Sullivan and Kay 1998; Ruiz Santiago et al. 2001; Shaw and Connor 2004).

## Conclusions

The levator claviculae is a source of controversy and yet to be fully explained. However, future molecular embryology will be able to provide a detailed description of the embryogenesis, and the genetics-based analyses will be able to explain homology across species. Advanced imaging techniques such as echography, CT, and MRI will also accurately elucidate the prevalence and the clinical importance of this unusual and rare muscle in humans.

Overall, we conclude that the levator claviculae shares a common embryological origin with the levator scapulae. In other words, the levator claviculae and the levator scapulae arise from the same myotome. This myotome also partially gives rise to both the trapezius and the sternocleidomastoid (McKenzie 1955), indicating the possibility that the levator claviculae fuses with those two muscles in a few rare cases (O’Sullivan and Kay 1998; Feigl and Pixner 2011).
